# Diastolic Blood Pressure Abnormalities and Their Relationship with Glycemic Control in Pediatric Type 1 Diabetes

**DOI:** 10.3390/jcm14134704

**Published:** 2025-07-03

**Authors:** Anna Stępniewska, Ewa Szczudlik, Dorota Drożdż, Joanna Nazim, Jerzy Starzyk, Dominika Januś, Małgorzata Wójcik

**Affiliations:** 1 Department of Pediatric and Adolescent Endocrinology, Chair of Pediatrics, Institute of Pediatrics, Faculty of Medicine, Jagiellonian University Medical College, 30-663 Cracow, Polandjoanna.nazim@uj.edu.pl (J.N.);; 2 Department of Pediatric Nephrology and Hypertension, Jagiellonian University Medical College, 30-663 Cracow, Poland; dorota.drozdz@uj.edu.pl

**Keywords:** arterial hypertension, ambulatory blood pressure monitoring, diabetes complications, metabolic control, HbA1c

## Abstract

**Background/Objectives**: Type 1 diabetes (T1D) in children is associated with increased cardiovascular risk, partly due to coexisting blood pressure (BP) disturbances. Ambulatory blood pressure monitoring (ABPM) is recommended for detecting subtle BP abnormalities, yet the relationship between glycemic control, T1D duration, and specific BP disturbances remains unclear. This study evaluated associations between HbA1c levels, T1D duration, and ABPM-derived BP parameters in a pediatric population with T1D. **Methods**: We included 357 children and adolescents (aged 7–18.8 years) with T1D treated at a tertiary center. All participants underwent 24 h ABPM. Glycemic control was assessed using HbA1c; values > 6.5% were considered suboptimal. We analyzed associations between HbA1c, T1D duration, and various BP parameters, including daytime and nighttime systolic and diastolic BP, nocturnal dipping, and hypertension defined by ABPM criteria. Logistic regression analyses were performed to identify independent predictors of elevated HbA1c. **Results**: Arterial hypertension was confirmed in 10% of patients, and 41% showed a non-dipping BP profile. There were no significant differences in HbA1c or T1D duration between dippers and non-dippers. However, patients with HbA1c > 6.5% had significantly higher 24 h diastolic BP and were more likely to meet hypertension criteria (*p* = 0.009). In univariate regression, both longer T1D duration (OR = 1.086; *p* = 0.033) and higher 24 h diastolic BP (OR = 1.065; *p* = 0.0068) were associated with elevated HbA1c. Both remained significant in multivariate analysis. **Conclusions**: Impaired glycemic control in children and adolescents with T1D was independently associated with higher 24 h diastolic BP and longer diabetes duration.

## 1. Introduction

Diabetes mellitus type 1 (T1D) is one of the major risk factors for the development of premature vascular aging, arterial hypertension (AH), and eventually cardiovascular impairment [1,2,3]. In children with T1D and AH, coexistence results in a 2–10 times greater risk for cardiovascular morbidity and mortality in adulthood [3,4,5]. Monitoring T1D children for the presence of AH is therefore of great clinical importance. However, standard office blood pressure (BP) measurements may miss the diagnosis, as visualized by the broad range of AH prevalence reported in the literature from 6% to 51% depending on the diagnostic methods used [6,7,8]. Additionally, office BP gives limited insight into BP abnormalities such as nocturnal or white coat hypertension, often complicating the right diagnosis. Therefore, 24 h ambulatory blood pressure monitoring (ABPM) has become the gold standard for detecting AH in children and adolescents with T1D [3,9,10]. ABPM also allows for detailed assessment of BP abnormalities during the day and night, such as day- and nighttime systolic and diastolic BP, nighttime blood pressure dip, or BP loads [3,9,11]. Abnormal nighttime BP profiles have been found to have significant prognostic value for predicting nephropathy and other vascular complications in T1D [12,13]. Physiological decline in BP during sleep typically ranges between 10 and 30% [14,15,16]. Characterized by a reduction of less than 10%, the non-dipping physiology is considered an early marker of increased sympathetic nervous system activation and increased cardiovascular risk [14,17,18]. The prevalence of the non-dipper profile among individuals with T1D ranges from 37% to 48% [19,20]. However, the relationship between disease duration, metabolic control, and nighttime BP abnormalities remains insufficiently understood, with limited and sometimes conflicting data [19,20,21]. This study aimed to evaluate the association between glycemic control, T1D duration, and BP disturbances assessed using ABPM in a large cohort of children and adolescents with T1D. Identifying risk factors for AH and abnormal BP patterns could support earlier intervention strategies to mitigate long-term cardiovascular complications.

## 2. Materials and Methods

### 2.1. Study Group

We included in this single-center study all consecutive children and adolescents with T1D treated in the tertiary pediatric endocrinology center. Age between 5 and 18, confirmed diagnosis of T1D, and ≥3 months of insulin therapy were the inclusion criteria. Exclusion criteria were any history of AH diagnosis, current or former antihypertensive treatment, a history of lipid abnormalities or lipid-lowering medications, and a history of any thyroid disease.

Written informed consent was obtained from all participants and their legal guardians. The study was approved by the Institutional Ethics Committee at Jagiellonian University in Krakow (KBET/94/B/2006), Poland, and was performed in accordance with the Declaration of Helsinki.

### 2.2. Ambulatory Blood Pressure Monitoring

Every participant underwent ABPM using a validated oscillometric device (Spacelabs 90217, Issaquah, WA, USA) suitable for children over 5 years of age and ≥120 cm in height. Cuff size was selected based on an individual measurement of non-dominant arm circumference. The following ABPM parameters were recorded: mean daytime and nighttime systolic blood pressure (SBP) and diastolic blood pressure (DBP), mean 24 h systolic and diastolic BP, mean 24 h arterial pressure, mean 24 h systolic and diastolic BP loads calculated as a % of readings above the 95th percentile, and the nocturnal dipping calculated using the formula recommended by the American Heart Association (AHA):

Dipping = [(mean daytime BP − mean nighttime BP)/mean daytime BP] × 100, and expressed as %. A normal dip was defined as ≥10% reduction [9]. Non-dipping was defined as <10% reduction in SBP and/or DBP [22]. AH was defined according to the 2014 AHA criteria as mean 24 h SBP and/or DBP ≥ 95th percentile for age, sex, and height [23].

### 2.3. Metabolic Control and T1D Duration

We used the glycated hemoglobin (HbA1c) value for the assessment of glycemic control. Favorable control was defined as HbA1c ≤ 6.5% and insufficient as HbA1c > 6.5% by the Polish Diabetes Association guidelines [24]. To define disease duration, we used the 5-year time (<5 yr and ≥5 yr) from diagnosis to study inclusion in line with previous studies [19,25,26].

The primary objective of this study was to assess the relation between glycemic control and the presence of nighttime BP disturbances such as mean nighttime SBP, DBP, and nocturnal dipping. The secondary objectives included evaluating the association between metabolic control and T1D duration and the presence of AH, day- and nighttime SBP, DBP, mean 24 h systolic and diastolic BPs, mean arterial pressure, and BP loads.

### 2.4. Statistical Analysis

Statistical analysis was performed using Statistica software, version 14.1.0.4 (1984–2023 Cloud Software Group). Continuous variables were expressed as means ± standard deviations (SDs). Categorical variables were presented as counts and percentages. Comparisons between groups were performed using the unpaired Student’s t-test for normally distributed parameters. The chi-square test was used for categorical data. Univariate and multiple stepwise linear regression analysis with the use of a manual stepwise variable elimination method was used to adjust the association between HbA1c ≤ 6.5% >6.5% and BP disturbances for possible confounders such as age, gender, BMI, and T1D duration. A *p*-value < 0.05 was considered statistically significant.

## 3. Results

### 3.1. Patient Characteristics

There were 357 patients (171 girls), aged 7–18.8 years (mean 16 ± 2 years), included in the study. Details are presented in Table 1. The mean T1D duration was 6.9 ± 3.5 years, ranging from 1 to 16.5. There were 245 (69%) patients with a T1D duration of >5 years, with similar distribution among girls and boys [120 (49%) vs. 125 (51%), *p* = 0.6, respectively]. The mean HbA1c was 7.6 ± 1.5%. There were 281 (79%) with HbA1c > 6.5%, with no differences between girls and boys [138 (50%) vs. 138 (50%), *p* = 0.2, respectively]. No patient had a diagnosis of hypertensive nephropathy, retinopathy, or neuropathy and cardiac disease.

### 3.2. Ambulatory Blood Pressure Monitoring Results

The rate of successful ABPM readings was 89.5 ± 9.1%. The ABPM results are shown in Table 2. AH based on ABPM criteria was confirmed in 36 (10%) patients [18 (50%) girls and 18 (50%) boys, *p* = 0.8]. The mean nocturnal dipping value was 10.7 ± 5.6%. There were 148 (41%) patients [75 (51%) girls, 73 (49%) boys, *p* = 0.4] with nighttime dipping of <10%.

### 3.3. Associations Between BP Disturbance, Glycemic Control, and T1D Duration

**Figure 1:**
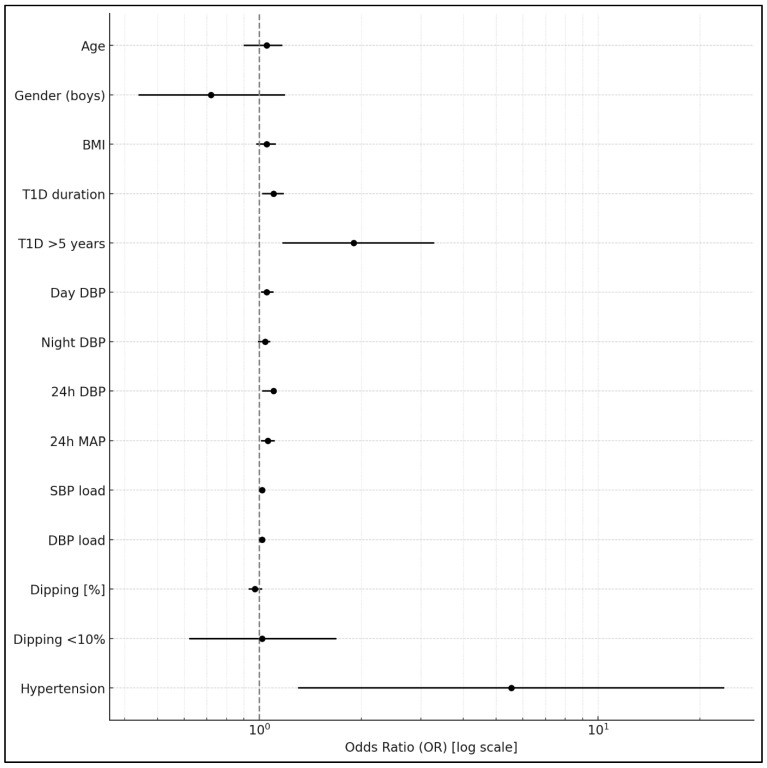
Association between clinical and blood pressure parameters and HbA1c > 6.5% in univariate logistic regression analyses. BMI: body mass index; T1D: type 1 diabetes mellitus; DBP: diastolic blood pressure; MAP: mean arterial blood pressure; SBP: systolic blood pressure.

There were no significant differences in HbA1c and T1D duration between patients with abnormal nocturnal dipping < 10% and normal dipping ≥10% (7.6 ± 1.6% vs. 7.6 ± 1.5%, *p* = 0.9, and 7.1 ± 3.6 years vs. 6.9 ± 3.5 years, *p*= 0.7, respectively) (Table 1). Patients with poor glycemic control and HbA1c > 6.5% had a worse diastolic BP profile, as shown in Table 3, compared to those with HbA1c of ≤6.5%. They were also more often diagnosed with AH [34 (12.4%) vs. 2 (2.5%), *p* = 0.009]. In univariate logistic regression, longer T1D duration (OR = 1.086; 95% CI: 1.007–1.172; *p* = 0.033) and higher 24 h diastolic BP (OR = 1.065; 95% CI: 1.018–1.115; *p* = 0.0068) were significantly associated with HbA1c > 6.5% (Table 4) (Figure 1). The presence of AH was also linked to impaired glycemic control (OR = 5.55; 95% CI: 1.30–23.62; *p* = 0.020). In the multivariate logistic regression analysis, both the 24 h diastolic BP and T1D duration remained independently associated with HbA1c > 6.5%.

## 4. Discussion

Our findings show that both poor metabolic control (HbA1c > 6.5%) and a longer duration of T1D are independently associated with higher 24 h DBP. We also found that impaired glycemic control had a significant impact on the presence of AH, as defined by 24 h ABPM. These observations support the growing body of evidence that metabolic control in pediatric T1D patients has important implications for vascular health, even early in life.

Our results are in line with previous studies that have reported associations between poor glycemic control and elevated blood pressure in young people with T1D [10,11,25]. Marcovecchio et al. demonstrated that higher HbA1c levels were associated with increased 24 h and nocturnal BP values in adolescents with T1D, suggesting early vascular dysfunction related to hyperglycemia [25]. Similarly, Lurbe et al. reported that elevated BP in adolescents with T1D may precede microvascular complications and could be related to metabolic abnormalities [1]. In our study, we were able to further identify 24 h diastolic BP as the BP parameter most strongly and independently associated with HbA1c levels, rather than systolic BP. This novel observation may suggest an early diastolic pattern of vascular stiffening or autonomic imbalance linked to poor metabolic control.

Unlike several previous reports [19,20,21], we did not find a significant association between nocturnal dipping patterns and either HbA1c or T1D duration. The relatively high prevalence of non-dipping (41%) in our cohort was consistent with other studies [16,19,20], but its lack of correlation with metabolic status suggests that other factors, such as pubertal stage, sleep quality, or autonomic regulation, may play a more dominant role in nocturnal BP regulation.

Patients with poor glycemic control in our study had significantly higher diastolic BP loads over 24 h, during the day and at night, compared to those with HbA1c ≤ 6.5%. These findings are consistent with previous reports suggesting that increased BP load, particularly diastolic, may be an early indicator of subclinical cardiovascular stress in children with T1D [11,25,26]. Our results extend this evidence by showing that elevated BP loads may also be linked to metabolic control in this population.

The observed association between poor glycemic control and the presence of hypertension is clinically relevant. Children with HbA1c > 6.5% had over fivefold increased odds of being diagnosed with AH. Although the wide confidence interval around this estimate reflects the relatively low number of hypertensive patients in our sample, this result highlights the potential role of metabolic factors in the development of early-onset hypertension. It also underscores the value of ABPM in detecting subclinical BP disturbances that may go unnoticed in office measurements.

Taken together, these findings reinforce the importance of regular ABPM assessment in children and adolescents with T1D, particularly those with a longer disease duration or elevated HbA1c. Early identification of subclinical BP abnormalities may provide an opportunity for timely intervention, potentially reducing long-term cardiovascular risk.

## 5. Limitations

Several limitations should be acknowledged. First, the cross-sectional design of this study does not allow for inference of causality between glycemic control and BP abnormalities. Second, although the sample size was relatively large, the number of children diagnosed with arterial hypertension was modest (n = 36), which may have limited the precision of effect estimates and resulted in wide confidence intervals. Third, we did not include data on pubertal stage, lipid profiles, insulin dose, or autonomic function, which are factors that could influence BP regulation. Finally, the absence of an age-matched healthy control group limits our ability to determine whether the observed blood pressure patterns reflect true pathology or fall within age-related variation. However, we addressed this by adjusting for potential confounders, including age, gender, BMI, and T1D duration, in multivariate regression models examining the relationship between HbA1c categories and BP disturbances.

## Figures and Tables

**Table 1:** Clinical characteristics of study patients and comparison between nocturnal non-dippers vs. nocturnal dippers.

Parameter	All Patients N = 357 Mean ± SD/n (%)	Abnormal Nocturnal Dipping of <10% N = 148 (41%) Mean ± SD/n (%)	Normal Nocturnal Dipping of ≥10% N = 209 (59%) Mean ± SD/n (%)	*p*-Value
Girls	172 (49%)	75 (50.7%)	97 (46.4%)	0.4
Age [years]	16.1 ± 5.7	16.2 ± 2	16.2 ± 7.1	0.9
Weight [kg]	65.5 ± 15.3	65.8 ± 14.9	65.3 ± 15.7	0.7
Height [cm]	168.5 ± 12.1	168.9 ± 11.1	168.3 ± 12.7	0.6
BMI [kg/m^2^]	22.7 ± 3.8	22.9 ± 3.6	22.6 ± 4.0	0.5
HbA1c [%]	7.6 ± 1.5	7.6 ± 1.6	7.6 ± 1.5	0.9
HbA1c > 6.5%	276 (77%)	114 (77%)	162 (71%)	0.9
T1D duration [years]	6.9 ± 3.5	7.1 ± 3.6	6.9 ± 3.5	0.7
T1D > 5 years	245 (69%)	103 (70%)	142 (70%)	0.7

BMI: body mass index; HbA1c: glycated hemoglobin; T1D: type 1 diabetes mellitus.

**Table 2:** Results of ambulatory blood pressure monitoring.

Parameter	Value Mean ± SD/n (%)
Mean day SBP [mmHg]	120.5 ± 12.5
Mean day DBP [mmHg]	70.9 ± 5.8
Mean night SBP [mmHg]	107.5 ± 13.5
Mean night DBP [mmHg]	58.8 ± 6.9
Mean 24 h SBP load [%]	16.7 ± 18.1
Mean 24 h DBP load [%]	15.2 ± 13.3
Nocturnal dipping [%]	10.7 ± 5.6
Nocturnal dipping < 10%	148 (41%)
AH	36 (10%)

SBP: systolic blood pressure; DBP: diastolic blood pressure; AH: arterial hypertension.

**Table 3:** Associations between ambulatory blood pressure monitoring parameters, glycemic control, and T1D duration.

Parameter	HbA1c Mean ± SD/n (%)		*p*-Value	T1D Duration Mean ± SD/n (%)		*p*-Value
	≤6.5% N = 81 (23%)	>6.5% N = 276 (77%)		≤5 Years N = 112 (31%)	>5 Years N = 245 (69%)	
Mean day SBP [mmHg]	118.5 ± 14.1	121 ± 12	0.1	119.4 ± 13.4	120.9 ± 12.1	0.3
Mean day DBP [mmHg]	69.6 ± 6.1	71.4 ± 5.7	0.01	70.5 ± 6.2	71.2 ± 5.6	0.3
Mean night SBP [mmHg]	105.1 ± 14.8	108.1 ± 13.7	0.08	105.8 ± 16.5	108.1 ± 11.9	0.1
Mean night DBP [mmHg]	57.1 ± 8.7	59.3 ± 6.4	0.01	57.8 ± 8.2	59.3 ± 6.3	0.06
Mean 24 h SBP [mmHg]	117.1 ± 8.2	122.5 ± 60.7	0.4	126.7 ± 94.6	118.8 ± 9.6	0.2
Mean 24 h DBP [mmHg]	66.4 ± 8.7	69 ± 5.3	0.0009	67.4 ± 8.1	68.9 ± 5.2	0.04
Mean 24 h arterial pressure [mmHg]	84.3 ± 5.2	86.1 ± 5.7	0.01	85.2 ± 5.6	86 ± 5.7	0.2
Mean SBP load [%]	12.1 ± 14	18 ± 18.9	0.01	14.9 ± 18.5	17.5 ± 17.8	0.2
Mean DBP load [%]	12.5 ± 12.4	16 ± 13.4	0.03	14.1 ± 13.9	15.8 ± 13	0.2
Nocturnal dipping [%]	11.4 ± 5.6	10.6 ± 5.7	0.2	10.8 ± 5.8	10.8 ± 5.6	0.9
Nocturnal dipping < 10%	34 (42%)	114 (41%)	0.9	45 (40.2%)	103 (42%)	0.7
Arterial hypertension	2 (2.5%)	34 (12.4%)	0.009	12 (10.7%)	24 (9.8%)	0.8

SBP: systolic blood pressure; DBP: diastolic blood pressure; AH: arterial hypertension.

**Table 4:** Associations between BP disturbances and HbA1c ≤ 6.5% >6.5%.

Variable	Univariate Logistic Regression		Multivariate Logistic Regression (R^2^ = 0.06)	
	OR (95% CI)	*p*-Value	OR (95% CI)	*p*-Value
Age [years]	1.05 (0.9–1.17)	0.3		
Gender [0—girls; 1—boys]	0.72 (0.44–1.19)	0.2		
BMI [kg/m^2^]	1.05 (0.98–1.12)	0.13		
T1D duration [years]	1.1 (1.02–1.18)	0.01	1.09 (1.01–1.17)	0.03
T1D [0–≤5 years 1–>5 years]	1.9 (1.17–3.28)	0.01		
Mean day DBP [mmHg]	1.05 (1.01–1.1)	0.01		
Mean night DBP [mmHg]	1.04 (0.99–1.08)	0.06		
Mean 24 h DBP [mmHg]	1.1 (1.02–1.12)	0.003	1.07 (1.02–1.12)	0.007
Mean 24 h arterial pressure [mmHg]	1.06 (1.01–1.11)	0.01		
Mean SBP load [%]	1.02 (1.0–1.04)	0.01		
Mean DBP load [%]	1.02 (1.0–1.04)	0.04		
Nocturnal dipping [%]	0.97 (0.93–1.02)	0.3		
Nocturnal dipping [0–<10%; 1–≥10%]	1.02 (0.62–1.69)	0.9		
Arterial hypertension [0—absent; 1—present]	5.55 (1.3–23.6)	0.02		

BMI: body mass index; HbA1c: glycated hemoglobin; T1D: type 1 diabetes mellitus; SBP: systolic blood pressure; DBP: diastolic blood pressure; AH: arterial hypertension.

## Data Availability

Data can be provided upon request.
